# Microwave Treatment of Calcium Phosphate/Titanium Dioxide Composite to Improve Protein Adsorption

**DOI:** 10.3390/ma15144773

**Published:** 2022-07-07

**Authors:** Kyung Hee Park, Ho-Jun Song, Yeong-Joon Park

**Affiliations:** Department of Dental Materials and Hard-Tissue Biointerface Research Center, School of Dentistry, Chonnam National University, Gwangju 61186, Korea; see0936@jnu.ac.kr (K.H.P.); songhj@jnu.ac.kr (H.-J.S.)

**Keywords:** TiO_2_ nanoflower, bovine serum albumin, protein adsorption, calcium phosphate, microwave treatment

## Abstract

Calcium phosphate has attracted enormous attention as a bone regenerative material in biomedical fields. In this study, we investigated the effect of microwave treatment on calcium phosphate deposited TiO_2_ nanoflower to improve protein adsorption. Hierarchical rutile TiO_2_ nanoflowers (TiNF) fabricated by a hydrothermal method were soaked in modified simulated body fluid for 3 days to induce calcium phosphate (CAP) formation, followed by exposure to microwave radiation (MW). Coating the dental implants with CAP/TiNF provides a means of improving the biological properties, as the structure, morphology, and thickness of the composites can be controlled. The composites were characterized by scanning electron microscopy (SEM), X-ray diffraction (XRD), field emission transmission electron microscopy (TEM), and Fourier-transform infrared spectroscopy (FTIR), respectively. The composites were identified to be composed of aggregated nano-sized particles with sphere-like shapes, and the calcium phosphate demonstrated low crystallinity. The ability of bovine serum albumin (BSA) to adsorb on MW-treated CAP/TiNF composites was studied as a function of BSA concentration. The Sips isotherm was used to analyze the BSA adsorption on MW-treated CAP/TiNF composites. The MW-treated samples showed high protein adsorption capacity, thereby indicating their potential in various biomedical applications.

## 1. Introduction

Calcium phosphate (CAP) has been used in medicine and dentistry for a long time because of its excellent biocompatibility, bioactivity, and osteoconduction characteristics [1]. CAP ceramics are more biocompatible than other materials that are used for hard tissue replacement due to their close resemblance with living tissue in terms of composition. However, such materials have not been widely investigated concerning protein adsorption, and the effect on cell adhesion is not clear [2]. Among various surface properties, surface roughness and composition are generally considered the most important parameters that alter cell activity. Surface treatment of the ceramic materials is necessary to produce microstructures with the required properties. Sintering affects the properties of the materials, such as chemical composition and microstructure, which influences the biological and mechanical performance of CAP ceramics [3,4,5].

Fang et al. first reported microwave (MW)-sintering of calcium hydroxyapatite using a 700 W, 2.45 GHz microwave oven [6]. MW treatment resulted in materials with high density, better microstructure, and greater hydroxyapatite (HA) strength within a relatively short period of time compared to heating in a conventional furnace. Most previous microwave sintering studies found that MW treatment not only renders the materials more osteo-mimetic and biocompatible but also improves their mechanical strength [7]. The obvious advantages of MW sintering technology in terms of energy-saving and improvement in the mechanical properties of ceramics have made this technology popular in recent years, especially in the field of bioceramics [8].

Titanium oxide (TiO_2_) has gained interest as a coating system on Ti. The biocompatibility of Ti is attributed to the existence of a thin TiO_2_ layer on the surface, which is easily formed under moist conditions [9]. For the TiO_2_ layer to be effective in the presence of body fluids, it should be thick enough to prevent corrosion of the Ti surface. Therefore, several different methods of promoting the formation of the TiO_2_ layer on Ti were developed. The hydrothermal technique is one of the most convenient and effective methods for the preparation of titanium dioxide nanostructures. Arrays of TiO_2_ nanotubes are used as an efficient means of overcoming the problem of low interfacial bonding of CAPs coatings [10,11]. Previously, CAP/TiO_2_ nanoflowers (TiNF)-composites were prepared by immersing TiNF/Ti substrates in an m-SBF solution. The TiO_2_ nanostructures promote apatite formation to a greater extent than the common native oxide layer on titanium [12].

The biomaterial that is used during in vivo implantation or tissue engineering has an essential need for the immediate adherence of protein molecules in the blood and body fluids to continue cellular response, cell spreading, migration, and proliferation via the protein-coated layer [13,14]. Tissue engineering demands an understanding of the interactions between synthetic biomaterials and proteins, which strictly depend on the morphology and crystalline structure of the biomaterials. Recently, protein adsorption on the hydroxyapatite surface has been reported to significantly enhance cell adhesion, viability, and proliferation at the nano-biointerface [15].

BSA has an interesting biomedical application as it can interact with many organic and inorganic molecules and drugs due to the multiple binding sites on the exposed surface of the molecule. Detecting BSA protein by optical methods is cumbersome and labor-intensive, which therefore demands an alternative approach with an environmentally friendly, non-toxic, and easy detection method. Hence, it is important to explore the detection of BSA using a TiO_2_-CAP-based biosensor due to its low operating voltage and high sensitivity. Among various analytical methods developed for albumin protein detection, spectrophotometric methods are fast and highly sensitive. On binding the protein with the oxide surface, the optical properties of metal oxide-BSA composite undergo changes, and the same can be confirmed with the spectroscopic methods [16,17,18,19].

The present work aims to study MW treatment of CAP/TiNF composites with various exposure times and the effects of such treatment on the microstructure and surface properties of the composites. The influence of nanostructure morphology and crystallinity following microwave treatment on the adsorption of BSA is systematically addressed.

## 2. Materials and Methods

### 2.1. Preparation of TiO_2_ Nanomaterials

TiNF deposit on polished pure titanium substrates (cp-Ti, grade 2, 12 mm diameter) was achieved via a hydrothermal method, as described previously [12]. In short, 0.75 mL of titanium (IV) isopropoxide (TTIP, 97%, Sigma-Aldrich, St. Louis, MO, USA) as a precursor, 15 mL of hydrochloric acid (HCl, 37%, Merck, St. Louis, MO, USA), and 15 mL of double distilled water were mixed in a Teflon-lined autoclave vessel with a 50 mL capacity at room temperature. The temperature of the autoclave was set at 180 °C, and the growth of the nanoflower structure was achieved for 3 h with a heating rate of 5 °C min^−1^ and natural furnace cooling.

### 2.2. Preparation of Calcium Phosphate Coatings and Microwave Treatment

The preparation of simulated body fluid (SBF) and immersion of TiNF specimens in SBF solution was performed following the method described by Kokubo et al. [20]. The m-SBF solution was prepared by increasing the concentrations of CaCl_2_ and KH_2_PO_4_ twice as compared to those of the SBF solution to obtain more calcium phosphate (CAP). TiNF samples were immersed in 50 mL of modified-SBF (m-SBF) for 3 days at 37 °C. The m-SBF solution was changed after 2 days to maintain the concentration during the coating of calcium phosphate on TiNF. The m-SBF was prepared by dissolving NaCl, KCl, MgSO_4_, MgCl_2_, NaHCO_3_, CaCl_2_·2H_2_O, KH_2_PO_4_, and tris-hydroxymethyl aminomethane in ultra-pure water and titrating the solution to pH = 7.4 using 1 M HCl. The concentrations of inorganic ions (mM; Na^+^ 141.0, K^+^ 4.0, Mg^2+^ 1.5, Ca^2+^ 5, Cl^−^ 145.0, HCO_3_^2−^ 4.2, SO_4_^2−^ 0.5, and H_2_PO_4_^2−^ 2.0) were almost the same to the levels in human blood plasma. The chemical compositions of the SBF and m-SBFs are summarized in Table 1. The samples were taken out after 3 days, cleaned in deionized water, and dried at room temperature before characterization. Then, the CAP/TiNF films were treated in a commercial microwave oven (LG, ML39BW, 2.45 GHz) at 700 W for 5 to 15 min under an N_2_ atmosphere with a flow rate of 2 mL.

### 2.3. Characterization of CAP/TiNF Coatings

The surface morphologies of the TiNF and CAP/TiNF composites formed by immersion in m-SBF solution were recorded by field emission scanning electron microscopy (FE-SEM; S-4700, Hitachi, Tokyo, Japan). The crystalline structure of TiNF and MW-exposed CAP/TiNF nanostructures was characterized by the XRD patterns obtained using an XRD-6000 (Japan) X-ray diffractometer in the diffraction angle range of 5–80° with Cu-Kα radiation (40 kV, 30 mA, λ = 1.5406 Å). A step size of 0.04° and an accumulation time of 35 s were used. Field emission transmission electron microscopy (FE-TEM) images were obtained using JEM-2100F (Jeol Ltd., Tokyo, Japan) with a voltage of 200 kV. The FTIR spectra of the samples were recorded on an FTIR spectrometer equipped with an attenuated total reflection module (Tensor I, Bruker, Billerica, MA, USA) in the range of 4000–500 cm^−1^, with a resolution of 1 cm^−1^. The obtained spectra are the average of 32 scans. The wettability was evaluated by water contact angle measurements. The contact angle was obtained using the sessile drop method on a contact angle analyzer (Camscope, Sometech Co. Ltd., Seoul, Korea). Measurements were performed in triplicate.

### 2.4. Protein Adsorption

Bovine serum albumin (BSA, 99.8% purity, Sigma-Aldrich, St. Louis, MO, USA) (68 kDa, solubility 1 g in 25 mL of H_2_O) was prepared to a concentration of 10^−6^ mol L^−1^ BSA solution. The protein adsorption test was performed according to the standard procedure [21]. The BSA adsorption was assayed using the Pierce BCA Protein Assay Kit (Thermo Fisher Scientific, Inc., Waltham, MA, USA) in a 96-well plate. Briefly, the assay mixture contained 150 μL of the reagent (bicinchoninic acid solution, 4% (*w*/*v*) CuSO_4_·5H_2_O solution, and BSA protein standard solution), 150 μL of the sample containing BSA standard, and different potential interfering reagents. The protein adsorption experiments were performed at different protein concentrations as follows. MW-CAP/TiNF samples were soaked in 2 mL of BSA solution containing various concentrations of protein (0–1000 mg L^−1^). The sample-containing 96 well plate was placed in a shaking incubator at 37 °C for 30 min. Absorbance was measured immediately at 562 nm using a BioTek PowerWave H1 microplate spectrophotometer. Measurements were performed in triplicate. The maximum adsorption capacity of BSA (mg g^−1^) was plotted versus the equilibrium concentration (mg L^−1^). The data were fitted to the Langmuir and Sips models. The Langmuir and Sips equations are expressed as follows [22]:

(1) $$q_{e}=\frac{q_{m}b}{1+bC_{e}}$$

(2) $$q_{e}=\frac{q_{m}bC_{e}{}^{1/n}}{1+bC_{e}{}^{1/n}}=\frac{q_{m}bC_{e}{}^{m}}{1+bC_{e}{}^{m}}$$

where *q_e_* is the equilibrium amount of BSA adsorbed on an MW- TiNF-m3d coating layer (mg/g); *q_m_* is the maximum adsorption capacity of BSA (mg/g); *C_e_* is the concentration at equilibrium (mg/L); and *b* is the constant related to the affinity between BSA and the surface of MW-TiNF-m3d coating layer (L/mg); *n* is the Sips adsorption parameter; and m is the surface heterogeneity parameter. The adsorption curves were fit and graphed using the MATLAB R2021a (MathWorks, Natick, MA, USA) platform.

## 3. Results and Discussion

The flower-shaped TiO_2_ nanomaterials (TiNF) were obtained by hydrothermal treatment at 5:5 *v/v* ratio of hydrochloric acid to water for 3 h. Calcium phosphate deposits on TiNF (CAP/TiNF) were prepared by immersing in an m-SBF solution for 3 days. The CAP/TiNF samples were treated in a modified microwave oven for 5 to 15 min under an N_2_ atmosphere (shown in Supplementary Information).

**Figure 1:**
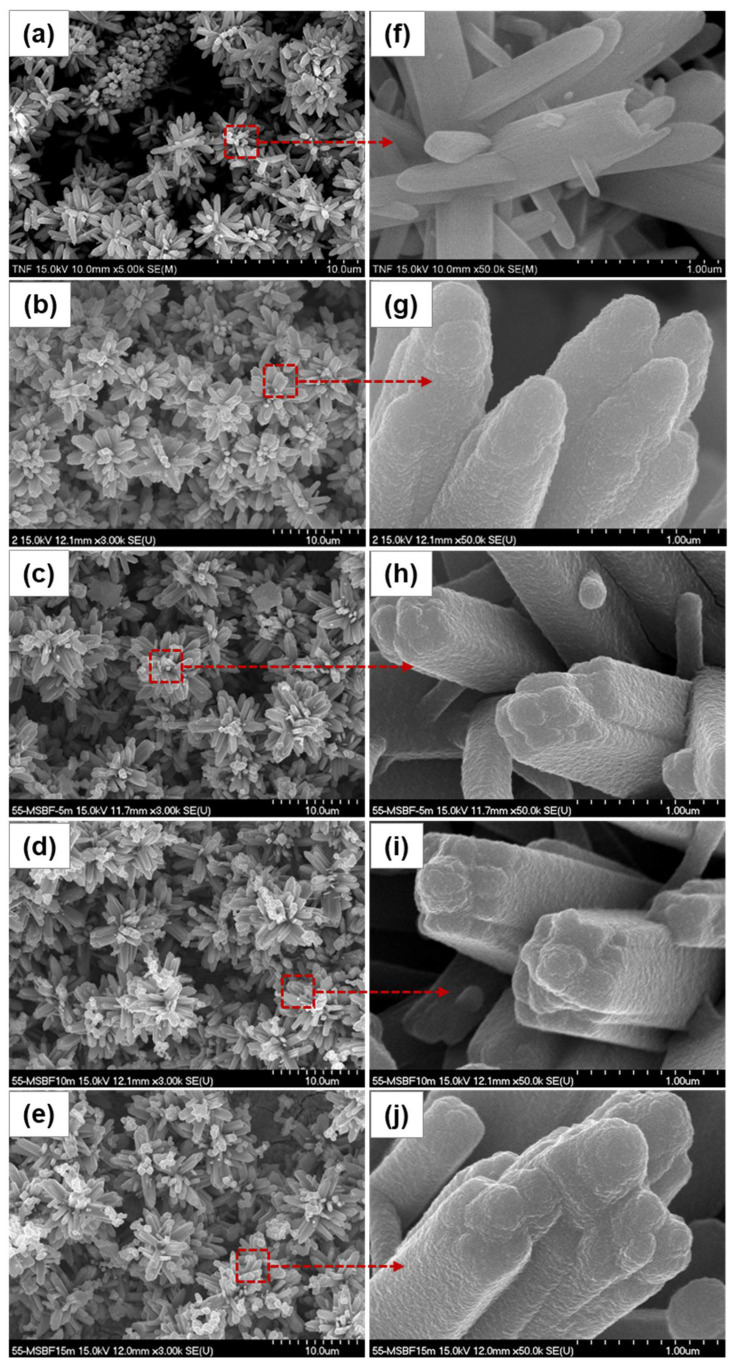
SEM images of CAP/TiNF composites prepared at different microwave exposure times: (**a**,**f**) TiNF, (**b**,**g**) 0 min, (**c**,**h**) 5 min, (**d**,**i**) 10 min, and (**e**,**j**) 15 min. Red-color dotted squares represent the formation of denser and globular particles on the CAP/TiNF surface after microwave treatment.

The morphologies of the TiNF and CAP/TiNF samples before and after MW treatment for different exposure times are shown in Figure 1. The synthesized TiNFs shown in Figure 1a consisted of numerous aligned nanorods with different orientations, thereby forming hierarchical flower-like nanostructures. Figure 1b displays the SEM images of CAP deposits on the surface of TiNFs after 3 days of immersion. Figure 1c shows the rapid agglomeration of small nanoparticles into compact microspheres with diameters of 300–400 nm. At an exposure time of 5 min, it appeared that a thick layer of dense CAP particles covered the entire TiNF surface. According to the SEM results, microwave treatment of the CAP/TiNF samples altered the morphology of the CAP particles. The CAP nanoparticles were round-shaped with diameters around 300 nm, and they tended to agglomerate on the edges of TiNFs (marked by the red circles) (Figure 1g–j). The morphological study revealed that most of the CAP nanoparticles were sphere-shaped and measured on the nanometer scale. Microwave treatment generates heat within the material first and then heats the entire volume. Zhou et al. reported that microwave irradiation is more effective in intensifying nucleation rather than accelerating crystal growth [23,24]. MW treatment resulted in the formation of inner TiNFs with tetragonal structures and obvious precipitation of spherical particles from the m-SBF solution onto the TiNF materials. With an increase in microwave exposure time, the nucleation and growth of the CAP particles on the edge of TiNFs continued. As a result, more and more globular particles were formed on the TiNF surface, and precipitation became denser (Figure 1j).

**Figure 2:**
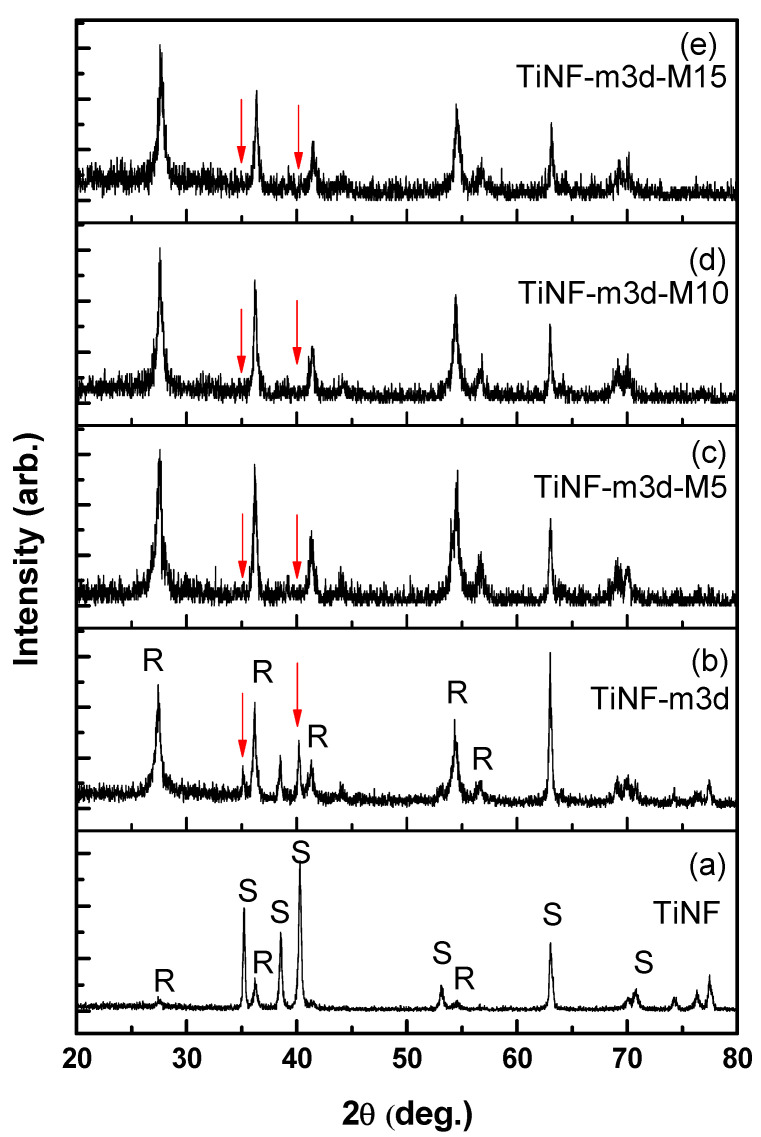
XRD patterns of TiNF and CAP/TiNF at different microwave exposure times; (**a**) TiNF before soaking in m-SBF solution, (**b**) TiNF after soaking in m-SBF solution for 3 days, and TiNF after soaking in m-SBF solution for 3 days followed by microwave treatment for (**c**) 5 min, (**d**) 10 min, and (**e**) 15 min, respectively. The positions of the substrate peaks are marked with red arrows. The peaks of rutile TiO_2_ and cp-Ti substrate are denoted as R and S, respectively.

The XRD patterns of TiNF and CAP/TiNF with different MW exposure times are shown in Figure 2. In Figure 2a, the XRD pattern of the TiNF sample confirmed the formation of the rutile TiO_2_ phase (JCPDS 89-4920) and cp-Ti substrate peak (marked with ‘S’). The spectrum of the CAP/TiNF precipitate formed without microwave exposure showed no presence of new peaks except the rutile TiNF peaks and substrate peaks (Figure 2b). In Figure 2c–e, several weak diffraction peaks were observed, which could not be assigned as peaks of CAP. The intensities of substrate peaks (red arrow) were decreased because TiNF/Ti surface was covered with nano-sized CAP particles. The broadening of XRD peaks of CAP was observed. Londoño-Restrepo et al. reported that broad X-ray peaks are related to the nanometric size of the particles [25]. The XRD patterns confirmed that an increase in microwave radiation time does not alter the crystalline structure of the material. We performed transmission electron microscopy analysis of the CAP/TiNF and MW-CAP/TiNF samples to confirm the formation of calcium phosphate nanocrystals.

**Figure 3:**
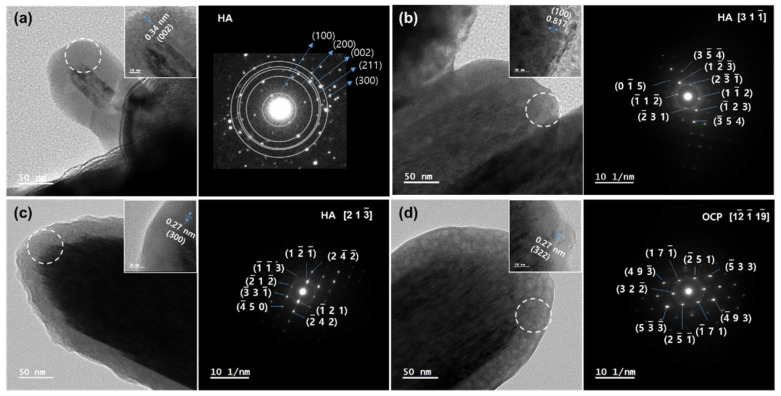
HRTEM images of CAP/TiNF composites formed at different microwave exposure times: (**a**) 0 min, (**b**) 5 min, (**c**) 10 min, and (**d**) 15 min. Inset refers to the high-resolution TEM images (HRTEM). Selected area electron diffraction (SAED) patterns of the highlighted area of the HRTEM images.

HRTEM images of the CAP/TiNF crystals are shown in Figure 3. As shown in Figure 3a, CAP exhibits a rod-shaped morphology of rutile TiNF. The square in Figure 3a represents the area of the high-resolution TEM lattice image. The SAED pattern of the CAP/TiNF composites indicates the polycrystalline nature of the sample. Major patterns were identified to (100), (200), (002), (211), and (300) planes of the hexagonal structure of HA. The HRTEM analysis revealed that the lattice fringe corresponding to the (002) plane was 0.34 nm. A selected area image of HRTEM is also shown as an inset in Figure 3. In Figure 3b,c, the SAED patterns taken from a single nanocrystal revealed that the particles were single crystals of hexagonal HA. The highly oriented diffraction patterns indicate the single crystalline property of the HA. The *d*-spacing of 0.82 nm obtained from the highly magnified TEM image was ascribed to the adjacent (100) plane of HA (Figure 3b). In Figure 3c, the lattice spacing was determined to be 0.27 nm corresponding to the (300) plane. In Figure 3d, the SAED pattern of OCP crystals indicates the reflections along the [12119] zone axis, as evidenced by the presence of diffraction patterns corresponding to *d*-spacing of 0.27 nm to the (322) plane, which are the characteristics of OCP. The HRTEM analysis of CAP/TiNF samples with microwave treatment for 0–10 min revealed the HA structure, but the microwave-treated CAP/TiNF sample (for 15 min) demonstrated the OCP structure.

**Figure 4:**
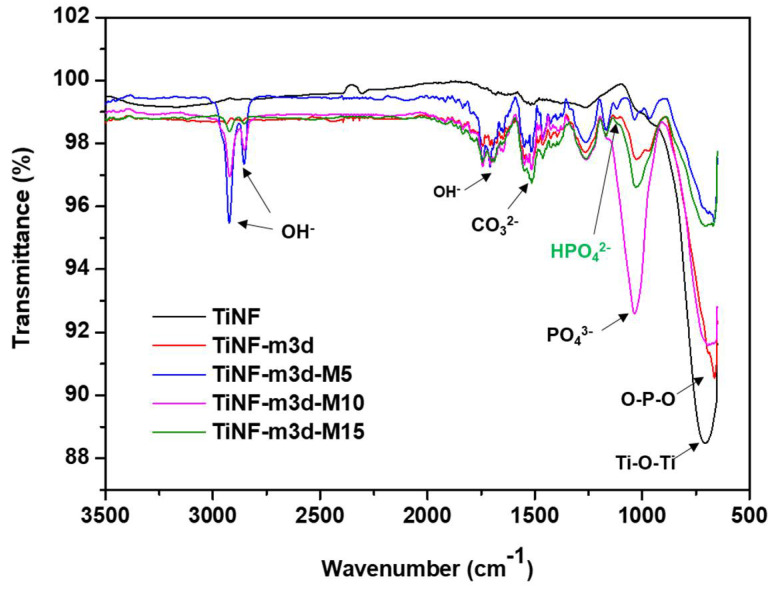
FT-IR spectra of TiNF and CAP/TiNF with different microwave exposure times. TiNF refers to hydrothermally synthesized TiO_2_ nanoflower, and TiNF-m3d is TiNF after soaking in m-SBF solution for 3 days. TiNF-m3d was subsequently microwave treated for 5, 10, and 15 min to make TiNF-m3d-M5, TiNF-m3d-M10, and TiNF-m3d-M15, respectively.

The FT-IR spectra of the TiNF and CAP/TiNF composites treated in an MW for different times are shown in Figure 4. In the TiNF sample, the bending vibration of OH^−^ was observed in the range of 1500–1650 cm^−1^. The absorption peak around 708 cm^−1^ shows the bending vibrations of O-Ti-O. The intensity of the peak at 1028 cm^−1^ increased significantly, thereby indicating that more P-O-P was present on the surface of MW-TiNF-m3d samples compared with TiNF-m3d [10]. Additionally, the MW-TiNF-m3d samples demonstrated characteristic bands at 970 cm^−1^ corresponding to ν1 symmetric P-O stretching mode of the PO_4_^3−^ group of HA. Two new peaks at 2920 and 2840 cm^−1^ were observed for OH^−^ functional group [26]. The bands at 1167 and 1127 cm^−1^ correspond to the components of the antisymmetric P–O stretching mode. The bands at 570 and 664 cm^−1^ were assigned to the components of the O–P–O bending mode of HA. In the TiNF-m3d-M15 sample, the vibration mode of ν3 PO_4_^3−^ at 1025 cm^−1^ and ν3 HPO_4_^2−^ stretching mode at 1104 cm^−1^ were considered as the spectrum of the OCP [27]. It was observed that a significant concentration of hydroxyl group remained in the structure, as evidenced by the intensity of the stretching and vibrational bands at 664 cm^−1^. The *ν*3 vibration mode of CO_3_^2−^ groups from m-SBF solution was also observed in the FT-IR spectra near 1461 and 1419 cm^−1^.

**Figure 5:**
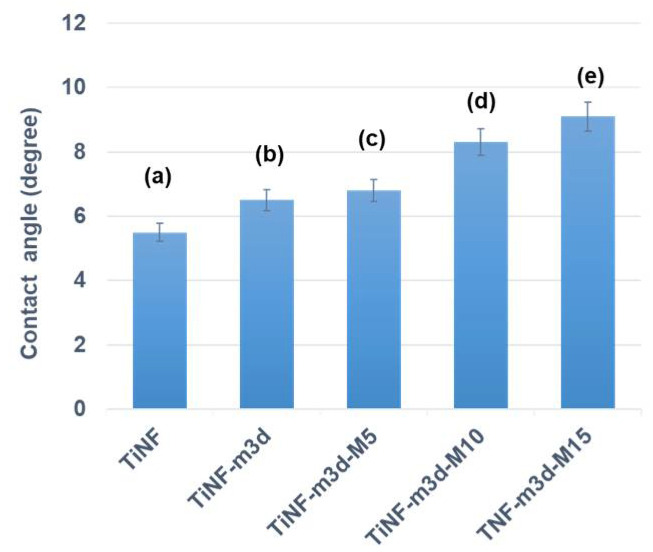
Water contact angles on (**a**) TiNF before immersing in m-SBF solution, (**b**) TiNF after immersing in m-SBF solution for 3 days, and TiNF after immersing in m-SBF solution for 3 days followed by MW treatment for (**c**) 5 min, (**d**) 10 min, and (**e**) 15 min, respectively.

Figure 5 shows the hydrophilic character of the MW-CAP/TiNF surface, which is a significant influencing factor in protein adsorption. The TiNF and CAP/TiNF samples are hydrophilic, with water contact angles of 5.5 ± 1.1° and 6.5 ± 0.5°, respectively. The contact angles of the MW-CAP/TiNF samples were slightly higher than that of untreated CAP/TiNF despite the fact that MW treatment induces high surface energy. The contact angle of TiNF-m3d-M5 was 6.8 ± 0.8°, and that of TiNF-m3d-M10 was 8.3 ± 0.7°, while that of TiNF-m3d-M15 reached 9.1 ± 0.5°; these angles showed similar values because there were only slight changes in the chemical structure of MW-CAP/TiNF samples. Therefore, the TiNF-m3d and MW-treated TiNF-m3d surfaces are both hydrophilic. Jeyachandran et al. observed that almost complete monolayer coverage of BSA adsorption could be achieved on a hydrophilic surface and that interactions with BSA molecules were much stronger on a hydrophilic than on a hydrophobic surface [28]. There are various possible reasons for the difference in water contact angle between the samples, but it was convincing that the primary factor associated with MW treatment is the alteration of surface functional groups. Therefore, SEM was employed to assess the change in surface roughness, and FT-IR analysis was performed to verify the change in O-H surface functional groups.

**Figure 6:**
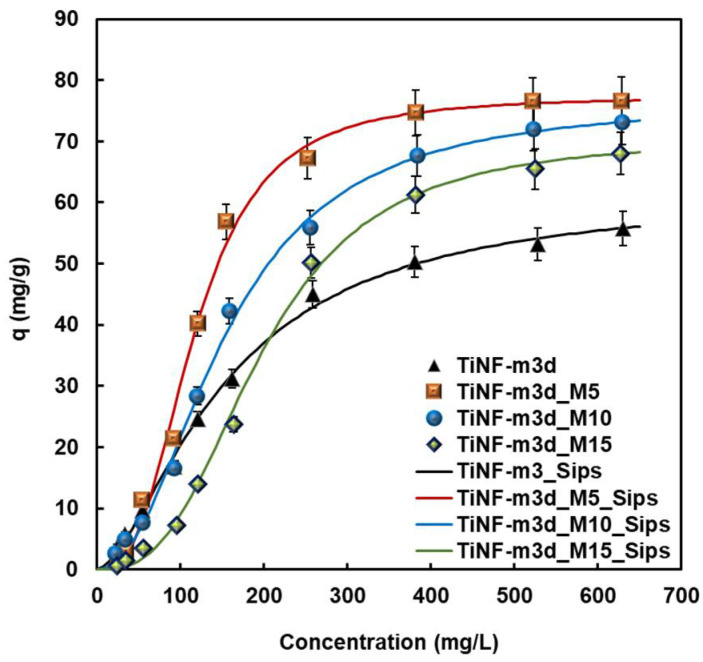
Adsorption isotherms of BSA on TiNF and CAP/TiNF coating layer with different MW exposure times. All measurements were made in triplicate.

Figure 6 shows the equilibrium data and isotherm of BSA adsorption on TiNF-m3d according to the MW exposure time at 37 °C. The adsorption phenomena of BSA on CAP nanoparticles is attributed to electrostatic interaction between Ca^2+^ and PO_4_^3−^ of calcium phosphate nanoparticles with COO^−^ and NH_4_^+^ of BSA, respectively [29]. The equilibrium of BSA adsorption on TiNF-m3d was well-fitted to the Sips model, and that of the MW-treated TiNF-m3d samples was also well-correlated to the Sips isotherm. Based on the maximum amount of adsorbed proteins (Table 2), the MW-treated TiNF-m3d films showed higher adsorption capacity than TiNF-m3d. This is because MW-treated TiNF films have a highly porous surface with a large degree of surface heterogeneity compared to TiNF-m3d film [30]. As shown in Figure 5, the adsorption isotherms have two regions before and after microwave exposure. At a low concentration of BSA, the first region was found to be unfavorable for the capillary condensation, while at high concentrations over 200 mg L^−1^, the second region was observed to be linear or favorable. The adsorption capacity decreased according to an increase in the MW exposure time in the low concentration range. MW treatment affects unfavorable adsorption at low concentrations to favorable surface adsorption due to the formation of a proteinaceous multilayer that participates in the electron transfer process.

Microwave treatment is advantageous due to a wide surface area exposure; hence has a greater possibility of interaction with cells and neighboring physiological environments. Microwave treatment is expected to have favorable biological effects based on the protein adsorption results of this work, and it shows a promising possibility for clinical application as orthopedic biomaterials. However, for the clinical application of this technique, cautious control against heat generation during the microwave treatment is necessitated.

## 4. Conclusions

The objective of the present work was to study the effects of MW treatment of CAP/TiNF on protein adsorption. Hierarchical rutile TiNF fabricated by the hydrothermal method was soaked in m-SBF for 3 days to encourage the formation of CAP, followed by exposure to MW radiation. The TiNF formed a rod-like morphology due to the stacking of the self-assembled spherical nanoparticles in a particular direction. These crystallites were aggregated with nano-sized particles that had spherical shapes and low HA crystallinity of calcium phosphate with MW exposure for 0~10 min. A slight increase in the contact angles was observed with an increase in MW exposure time. The TiNF-m3d and MW-treated TiNF-m3d surfaces were identified to be hydrophilic for BSA adsorption. The BSA adsorption capacity, which was correlated with the Sips isotherm of MW-treated TiNF-m3d, was greater than that of TiNF-m3d film due to the creation of a more porous surface with a relatively high degree of surface heterogeneity. As MW-treated CAP/TiNF demonstrates high protein adsorption capacity, it is proposed as a promising material for biomedical applications.

## Figures and Tables

**Table 1:** Chemical concentrations of standard SBF and modified SBF solutions.

Reagent	SBF		m-SBF	
	mM	g/L	mM	g/L
NaCl	141.0	8.240	141.0	8.240
KCl	4.0	0.300	4.0	0.300
MgSO_4_	0.5	0.060	0.5	0.060
MgCl_2_	1.0	0.950	1.0	0.950
NaHCO_3_	4.2	0.353	4.2	0.353
CaCl_2_·2H_2_O	2.5	0.368	5.0	0.736
KH_2_PO_4_	1.0	0.136	2.0	0.272
Tri-HCl		3.940		3.940

**Table 2:** Adsorption isotherm parameters for BSA adsorption on CAP/TiNF samples with different microwave exposure times.

Samples	Simulation Model	qm (mg g^−1^)	b (L mg^−1^)	m(-)	RMSE ^1^
TiNF-m3d	Langmuir Sips	91.97	2.93 × 10^−3^		0.973
		61.63	2.94 × 10^−3^	1.612	0.994
TiNF-m3d-M5	Sips	77.30	1.49 × 10^−6^	2.818	0.991
TiNF-m3d-M10	Sips	76.56	1.76 × 10^−5^	2.175	0.995
TiNF-m3d-M15	Sips	70.49	2.93 × 10^−7^	2.849	0.992

^1^ Root mean square error (RMSE): $\sqrt{\frac{1}{p-2}}\overset{p}{\underset{i=1}{\sum }}{\left(q_{t,meas}-q_{t,calc}\right)}^{2}$. Where *q_t,meas_* is adsorption capacity in the experiment (mg g^−1^), and *q_t,calc_* is the adsorption capacity calculated from models (mg g^−1^).

## Data Availability

The data presented in this study are available on request from the corresponding author.

## Supplementary Materials

The following supporting information can be downloaded at: https://www.mdpi.com/article/10.3390/ma15144773/s1.
