# Supplementary material for: Microwave Treatment of Calcium Phosphate/Titanium Dioxide Composite to Improve Protein Adsorption
Source: Materials (Basel). 2022 Jul 7;15(14):4773. doi: 10.3390/ma15144773 (PMC9316246; doi:10.3390/ma15144773)
Supplement: Supplementary file 1 [file materials-15-04773-s001.zip › materials-1773048-supplementary.pdf]

materials

MDPI

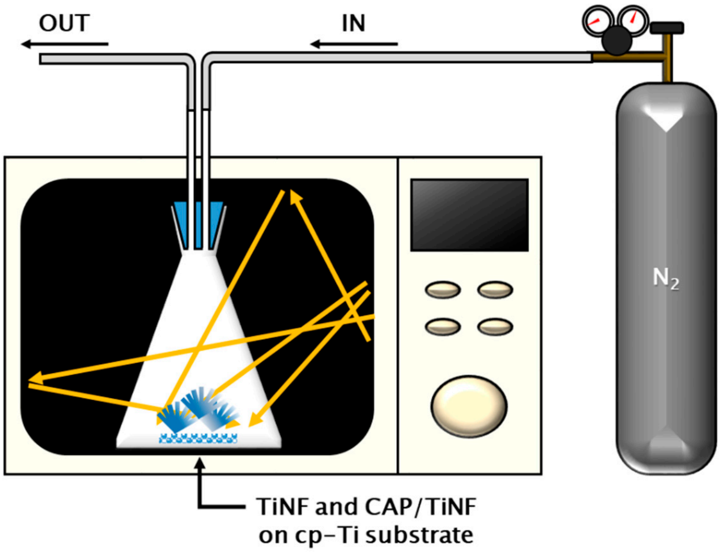

Figure S1. Schematic of the experimental set-up showing calcium phosphate/TiO_2 nanoflower sample preparation using microwave radiation.

The sample assemblies were placed in a modified commercial microwave (MW) oven (LG, ML39BW, 2.45 GHz, 700 W), and treated for 5 to 15 min under an N_2 atmosphere with a flow rate of 2 ml. MW oven was placed in a well-ventilated fume hood. The sample beakers were taken out and their contents were allowed to cool down to room temperature in an ambient atmosphere for the next 10–15 min.
